# The effect of cysteine oxidation on DJ-1 cytoprotective function in human alveolar type II cells

**DOI:** 10.1038/s41419-019-1833-5

**Published:** 2019-09-02

**Authors:** Karim Bahmed, Samia Boukhenouna, Loukmane Karim, Tessa Andrews, Jiusheng Lin, Robert Powers, Mark A. Wilson, Chih-Ru Lin, Elise Messier, Nichole Reisdorph, Roger L. Powell, Hsin-Yao Tang, Robert J. Mason, Gerard J. Criner, Beata Kosmider

**Affiliations:** 1 Department of Thoracic Medicine and Surgery, Temple University, Philadelphia, PA 19140 USA; 2 Center for Inflammation, Translational and Clinical Lung Research, Temple University, Philadelphia, PA 19140 USA; 3 University of Nebraska, Lincoln, NE 68588 USA; 4 Nebraska Center for Integrated Biomolecular Communication, University of Nebraska, Lincoln, NE 68588 USA; 5 Redox Biology Center, University of Nebraska, Lincoln, NE 68588 USA; 6 National Jewish Health, Denver, CO 80206 USA; 7 The Wistar Institute, Philadelphia, PA 19104 USA; 8 Department of Physiology, Temple University, Philadelphia, PA 19140 USA

**Keywords:** Mechanisms of disease, Respiratory tract diseases

## Abstract

DJ-1 is a multifunctional protein with cytoprotective functions. It is localized in the cytoplasm, nucleus, and mitochondria. The conserved cysteine residue at position 106 (Cys106) within DJ-1 serves as a sensor of redox state and can be oxidized to both the sulfinate (-SO_2_^−^) and sulfonate (-SO_3_^−^) forms. DJ-1 with Cys106-SO_2_^−^ has cytoprotective activity but high levels of reactive oxygen species can induce its overoxidation to Cys106-SO_3_^−^. We found increased oxidative stress in alveolar type II (ATII) cells isolated from emphysema patients as determined by 4-HNE expression. DJ-1 with Cys106-SO_3_^−^ was detected in these cells by mass spectrometry analysis. Moreover, ubiquitination of Cys106-SO_3_^−^ DJ-1 was identified, which suggests that this oxidized isoform is targeted for proteasomal destruction. Furthermore, we performed controlled oxidation using H_2_O_2_ in A549 cells with DJ-1 knockout generated using CRISPR-Cas9 strategy. Lack of DJ-1 sensitized cells to apoptosis induced by H_2_O_2_ as detected using Annexin V and propidium iodide by flow cytometry analysis. This treatment also decreased both mitochondrial DNA amount and mitochondrial ND1 (NADH dehydrogenase 1, subunit 1) gene expression, as well as increased mitochondrial DNA damage. Consistent with the decreased cytoprotective function of overoxidized DJ-1, recombinant Cys106-SO_3_^−^ DJ-1 exhibited a loss of its thermal unfolding transition, mild diminution of secondary structure in CD spectroscopy, and an increase in picosecond–nanosecond timescale dynamics as determined using NMR. Altogether, our data indicate that very high oxidative stress in ATII cells in emphysema patients induces DJ-1 overoxidation to the Cys106-SO_3_^−^ form, leading to increased protein flexibility and loss of its cytoprotective function, which may contribute to this disease pathogenesis.

## Introduction

Pulmonary emphysema is a life-threatening disease caused mainly by smoking^1–3^. It is associated with alveolar wall destruction and effective therapy against this disease is very limited. Alveolar type II (ATII) cells have stem cell potential, they proliferate and restore the epithelium after damage^4^. An imbalance between the excessive generation of reactive oxygen species (ROS) by cigarette smoke and their elimination via antioxidant defense systems can induce oxidative stress^5–7^. ROS can target and alter cellular macromolecules such as proteins, lipids, and DNA and contribute to lung diseases, including emphysema development^8,9^. ATII cells have a very high metabolism rate, therefore, their function largely depends on mitochondria^10^, which play a crucial role in energy production, metabolism, oxidative stress regulation, cell proliferation, and death^11^. ROS can damage mitochondrial proteins, leading to protein misfolding and aggregation. It has been shown that accumulation of oxidized and damaged mitochondrial proteins is implicated in lung diseases^12^.

DJ-1 is localized in the cytoplasm, nucleus, and mitochondria and can exert specific cytoprotective functions in different compartments^13^. It has been reported that this protein translocates to mitochondria under oxidative stress conditions and contributes to neuroprotection^14^. It is also involved in the regulation of mitochondrial dynamics by participation in oxidative stress response^15^. Human DJ-1 has three cysteine (Cys) residues: Cys46, Cys53, and Cys106^13^. Cys106 is the most reactive residue and allows DJ-1 to act as a sensor of cellular redox status^16,17^. Mutation of Cys106 to alanine (C106A) results in the loss of DJ-1 cytoprotective activity against oxidative stress^13,18^. Owing in part to its low pK_a_ value, Cys106 exists as the reactive thiolate^19^ that can be oxidized to either the sulfinate (-SO_2_^−^)^16,20^ or sulfonate (-SO_3_^−^) forms^17^. DJ-1 oxidizes readily to the Cys106-SO_2_^−^ form, which is a stable form and correlates with DJ-1’s cytoprotective activity^21^. However, DJ-1 overoxidation induced by very high oxidative stress causes modification of Cys106 to Cys106-SO_3_^−^^17^. This irreversible form has been suggested to inactivate DJ-1 function^22–24^; however, the mechanism is not well understood. Accumulation of Cys106-SO_3_^−^ DJ-1 has been correlated to diseases development where oxidative stress is a part of pathophysiology^25^. We have recently reported the cytoprotective role of DJ-1 against ATII cell injury in smokers^26^. Low DJ-1 levels and high DNA damage in ATII cells isolated from emphysema patients were observed^27,28^. Moreover, we reported that DJ-1 KO mice exposed to cigarette smoke had higher inflammatory response compared to wild-type mice. However, the molecular mechanisms of DJ-1 function in mitochondria and its response to oxidative stress in the lung remain largely elusive.

Here, we used human primary ATII cells obtained from control organ donors and emphysema patients, and report for the first time DJ-1 overoxidation in this disease. In addition, we found conformational changes of Cys106-SO_3_^−^ DJ-1, which can contribute to decreased DJ-1 cytoprotective function against cigarette smoke-induced oxidative stress.

## Materials and methods

### ATII cell isolation from control organ donors and emphysema patients

Lungs were obtained from organ donors not suitable for transplantation through the Gift of Life Foundation (Philadelphia, PA), the National Disease Research Interchange (NDRI) and the International Institute for the Advancement of Medicine (IIAM). Control non-smokers were individuals who never smoked and control smokers had a smoking history of 0.5–1 pack per day for at least 3 years. Lungs from patients with emphysema (GOLD 4) were obtained through Temple Biobank (Temple University, Philadelphia, PA). We used lung tissue from 3–7 individuals per group, 55–69-years-old, both females and males. We isolated ATII cells as we previously described^29^. Briefly, after instillation of 12.9 U/ml elastase (Worthington, Lakewood, NJ), the lung was minced followed by centrifugation to collect the cell suspension. The cells were filtrated and purified by a density gradient made of Optiprep (Accurate Chemical Scientific Corp., Westbury, NY). We used EpCAM (Miltenyi Biotec Inc, Auburn, CA) positive selection and the purity of isolated ATII cells was above 90% as determined using pro-SP-C antibody by flow cytometry analysis as we reported^29^. Subjects with emphysema provided written informed consent prior to surgery for use of these specimens and the relevant clinical and radiological data required for research. The study was performed in accordance with the Declaration of Helsinki and was approved by the Institutional Review Boards at Partners Healthcare and the Committee for the Protection of Human Subjects at Temple University.

### DJ-1 knockout cell line

The A549 cell line, which is a model for ATII cells, was maintained in Dulbecco’s modified Eagle medium (DMEM) supplemented with 10% fetal bovine serum (FBS) (both from Fisher Scientific, Hampton, NH) and 100 U/ml penicillin–streptomycin (GE Healthcare Life Sciences, Pittsburgh, PA). A549 cells with DJ-1 knockout (KO) were established using the CRISPR-Cas9 system and pX330 vector (Addgene, Watertown, MA), which encodes a Cas9 nuclease expression cassette and a guide RNA cloning cassette. Two types of DJ-1 guide RNAs targeting 5ʹ-AGTACAGTGTAGCCGTGATG-3ʹ and 3ʹ-TGCAAGCGCAAACTCGAAGC-5ʹ in exon 2 were selected using the CRISPR Design (crispr.mit.edu.) and ligated into the pX330 CRISPR Nuclease vector. A549 cells were transfected with generated pX330-RNAg vectors for 24 h. Cells were harvested and sorted for GFP-positive clones using FACS-VantageSE/DiVa cell sorter (BD, Franklin Lakes, NJ). Monoclonal A549 cell line with DJ-1 KO was validated by western blotting using DJ-1 antibody (Santa Cruz Biotechnology Inc., Dallas, TX). Growth of control A549 cells and A549 cells with DJ-1 KO was compared by plating 4 × 10^4^ cells per 1.9 cm^2^ on the plate and analyzed for 3 days.

Wild-type full-length DJ-1 sequence was cloned into pcDNA3.1 His-tag plasmid (Thermo Fisher Scientific, Waltham, MA). C106A mutant was generated by substitution of cysteine with alanine at position 106 by directed site mutagenesis. Plasmids were purified using Midi Prep Kit (Bioland Scientific, Paramount, CA). Transfection complexes were prepared using a ratio of 1:12 plasmid:polyethylenimine (PEI 25 K; Polysciences Inc., Warrington, PA) and incubated at room temperature for 15 min prior to transfection^30^. Cells were transfected with PEI 25 K complexes and plasmid and incubated at 37 ˚C for 24 h. Transfection efficiency was determined using mCherry-N1 expression plasmid (Clontech Laboratories, Inc., Mountain View, CA) by fluorescent microscope (Olympus, Center Valley, PA).

A549 cells and A549 cells with DJ-1 KO were treated with 1 mM H_2_O_2_ (Sigma, St. Louis, MO) as previously described^31–34^ for the indicated times to analyze DJ-1 oxidation. Cells were treated with 50 µM bleomycin (Cayman Chemicals, Ann Arbor, MI) for 2 h. Cell viability was determined using double staining with 0.01 mg/ml Hoechst 33342 and 0.001 mg/ml propidium iodide (PI; both from Sigma, St. Louis, MO). We analyzed 300 cells using fluorescence microscopy (Olympus, Center Valley, PA) in three independent experiments.

### Western blotting

Homogenized lung tissue or cell lysates were supplemented with phosphatase and protease inhibitors (both from Gold Biotechnology, Olivette, MO). Western blotting was performed as we previously described^26^. Briefly, 8–16% polyacrylamide gels (Invitrogen Corp, Carlsbad, CA) were used to separate the proteins. We used GAPDH (Abcam, Cambridge, MA), 4-HNE (Percipio Biosciences, Burlingame, CA), Cys106-oxidized DJ-1 (HCA024, Bio-Rad, Hercules, CA), *β*-actin (Sigma, St. Louis, MO), lamin-B1, Tom 20, DJ-1, His-tag, VDAC1, IKBα, COX IV and PDI (all were purchased from Santa Cruz Biotechnology, Dallas, TX). Horseradish peroxidase (HRP)-conjugated AffiniPure donkey IgG were obtained from Jackson ImmunoResearch (West Grove, PA). The blots were then developed using an enhanced chemiluminescence western blotting kit according to the manufacturer’s instructions (Amersham Pharmacia Biotech, Piscataway, NJ). Images were quantitated using ImageJ software.

### Oxidized glutathione (GSSG)/glutathione (GSH) measurement

A549 cells were treated with 1 mM of H_2_O_2_ for the indicated times. Following incubation, cells were washed with phosphate-buffered saline (PBS). The ratio of oxidized glutathione (GSSG) to total glutathione (GSH) was estimated using the Glo assay (Promega, Madison, WI) per manufacturer’s instructions.

### Mitochondrial DNA amount and mitochondrial DNA damage

Mitochondrial DNA (mtDNA) amount and mtDNA damage were assessed by qPCR and calculated as ratios of Ct _short mtDNA fragment_/Ct _short nuclear DNA fragment_ and Ct _long mtDNA fragment_/Ct _short mtDNA fragment,_ respectively^35,36^. Genomic DNA was isolated from A549 cells and DJ-1 KO A549 cells using EasyPrep Genomic DNA Mini Kit (Bioland Scientific, Paramount, CA). QPCR amplification was performed using SYBR Green Master Mix and StepOne Real-Time PCR System (Applied Biosystems, Foster City, CA) using the following conditions: 95 °C/10 min followed by 45 cycles of 95 °C/15 s, 58 °C/60 s, and 68 °C/150 s, prior to the melting curve 95 °C/15 s, 60 °C/15 s + 3% increment to 95 °C. A quantitative analysis of mtDNA amount and mtDNA damage are shown.

### Real-time PCR

Total RNA was isolated from A549 cells and A549 cells with DJ-1 KO using Quick-RNA MiniPrep (Zymo Research, Irvine, CA) according to the manufacturer’s recommendations. RNA was converted into complementary DNA (cDNA) using the SuperScript IV First-Strand Synthesis System (Invitrogen, Carlsbad, CA). DJ-1 and ND1 expression was determined by RT-PCR using SYBR Green Master Mix and StepOne Real-Time PCR System (Applied Biosystems, Foster City, CA) using the following conditions: 95 °C/10 min followed by 45 cycles of 95 °C/15 s, 58 °C/60 s, and 68 °C/20 s, prior to the melting curve 95 °C/15 s, 60 °C/15 s + 3% increment to 95 °C. Gene-specific primers were retrieved from PrimerBank (https://pga.mgh.harvard.edu/primerbank/) and ordered from Invitrogen (Carlsbad, CA). The sequences of the primers are provided in Supplementary Table 1. Relative fold changes of mRNA expression were calculated as a ratio to the expression of the reference gene, GAPDH using the 2^ddCt^ method.

### Subcellular fraction preparation

Subcellular fractions were prepared as previously described^37^. Briefly, A549 cells were resuspended in fractionation buffer containing 10 mM HEPES, 0.5 mM EGTA, 2 mM EDTA, 250 mM sucrose, 10 mM DTT, protease, and phosphatase inhibitor cocktail (Gold Biotechnology, Olivette, MO). Cell lysates were centrifuged at 1000 × *g* for 20 min. Supernatant was collected to isolate cytosolic, mitochondrial and endoplasmic reticulum (ER) fractions by sequential centrifugations and ultracentrifugation steps. The cell pellet was resuspended in a buffer composed of 20 mM HEPES, 400 mM NaCl, 1 mM EDTA, 1 mM EGTA, 1 mM DTT, protease, and phosphatase inhibitor cocktail and centrifuged at 14,000 rpm for 10 min to get the supernatant as a nuclear fraction. Lamin-B1 and IκB-α, COXIV, and PDI antibodies (Santa Cruz Biotechnology) were used to detect nuclear, cytosolic, mitochondrial, and ER fractions, respectively.

### Immunohistofluorescence

Localization of oxidized DJ-1 form was analyzed in cultured A549 cells treated with 1 mM H_2_O_2_ as described above. Cells were fixed with 4% paraformaldehyde, permeabilized with 0.2% Triton X-100 and blocked with 3% normal donkey serum (Jackson ImmunoResearch, West Grove, PA). Cells were incubated overnight with Cys106-oxidized DJ-1 (HCA024, Bio-Rad, Hercules, CA) and Tom20 (Santa Cruz Biotechnology, Dallas, TX) antibodies. The secondary antibodies, Alexa Fluor 488 and Alexa Fluor 594 IgG (Invitrogen, Carlsbad, CA) were applied for 1 h. The cells were then mounted with Vectashield medium containing DAPI (Abcam, Cambridge, MA) and analyzed using a fluorescence microscope (Olympus). We also analyzed DJ-1 oxidation in ATII cells using lung tissue obtained from non-smokers, smokers and emphysema patients. Sections were fixed in 4% paraformaldehyde (Electron Microscopy Sciences, Hatfield, PA), embedded in paraffin, deparaffinized and hydrated followed by antigen retrieval as we previously described^27^. SP-A (Novus, Biologicals, Littleton, CO) was used to identify ATII cells followed by incubation with Alexa Fluor 647 as described above.

### Mass spectrometry analysis

Immunoprecipitation was performed using DJ-1 antibody to analyze its oxidation status in freshly isolated ATII cells from control non-smokers, smokers, and patients with emphysema by mass spectrometry analysis. In the second approach, mitochondrial fractions obtained from cultured A549 cells were used to determine the protein content in bands at 23 kDa and 15 kDa using this method. A standard protein identification strategy was used for mass spectrometry analysis^38,39^. Trypsin digestion was applied to analyze MS/MS spectra (tandem mass spectrometry) generated from the LC-MS/MS runs (liquid chromatography with tandem mass spectrometry).

### Flow cytometry analysis

Viability of wild-type A549 cells and A549 cells with DJ-1 KO treated with H_2_O_2_ for 24 h was determined using Annexin V conjugated to Alexa Fluor 488 and 1 μg/ml propidium iodide (PI) (Thermo Fisher Scientific, Waltham, MA). Cells were trypsinized and washed with PBS prior to staining following the manufacturer’s instructions as we previously described^6^. Cell death was analyzed by LSR-II flow cytometer (BD Biosciences, San Jose, CA) and FlowJo (TreeStar). We also determined ROS generation using DCF-DA (2,7-dichlorofluorescein diacetate, Sigma, St. Louis, MO) probe. Briefly, A549 cells with DJ-1 KO were transfected with plasmid constructs (pcDNA3.1 empty vector, WT DJ-1, and C106A DJ-1) for 24 h. Cells were incubated with 10 µM DCF-DA for 45 min at 37 °C followed by treatment with 1 mM H_2_O_2_ or 50 µM bleomycin. The analysis was performed using LSR-II flow cytometer as described above.

### DJ-1 purification and controlled Cys106 oxidation

Unlabeled and uniformly ^15^N labeled human DJ-1 were expressed and purified as previously described^21,40^. Briefly, DJ-1 in pET15b (EMD Millipore, Darmstadt, Germany) was expressed by isopropyl β-d-1-thiogalactopyranoside (IPTG) induction in BL21(DE3) *Escherichia coli* (Novagen) grown in media supplemented with 100 μg/ml ampicillin at 37 °C. Luria-Bertani (LB) medium was used for the production of unlabeled protein and M9 minimal medium with 20% glucose and 0.1% (w/v) ^15^N NH_4_Cl (Cambridge Isotopes, Tewksbury, MA) was used to produce uniformly ^15^N labeled protein for nuclear magnetic resonance (NMR). DJ-1 was purified using Ni^2+^-NTA metal affinity resin (Sigma, St. Louis, MO) and the hexahistidine tag was removed using thrombin as previously reported^41^. DJ-1 ran as a single band in sodium dodecyl sulfate-polyacrylamide gel electrophoresis (SDS-PAGE) and was stored in storage buffer (25 mM HEPES-NaOH pH 7.5, 100 mM KCl, 3 mM dithiothreitol (DTT)).

To oxidize DJ-1 to the Cys106-SO_3_^−^ form, DTT in the storage buffer was removed by dialysis against storage buffer. H_2_O_2_ (30%, Thermo Fisher Scientific, Waltham, MA) was added to a final molar ratio of 100:1 H_2_O_2_:DJ-1 monomer and the sample was incubated at room temperature for 2.5 h. Unreacted peroxide was removed by centrifugal desalting using P6-DG resin (Bio-Rad, Hercules, CA). The oxidation state of the protein was confirmed by both intact mass and trypsinization electrospray mass spectrometry in the Nebraska Redox Biology Center Mass Spectrometry Core Facility. Mass spectrometry confirms that Cys106 is oxidized to the –SO_3_^−^ and that the other two cysteine residues (C46 and C53) are <5% oxidized.

### NMR relaxation measurements of DJ-1

Two-dimensional (2D) ^1^H-^15^N heteronuclear single quantum coherence (HSQC) spectra were collected at 37 °C as previously described^42,43^ from uniformly ^15^N labeled DJ-1 that was either reduced or overoxidized to Cys106-SO_3_^−^ The 2D ^1^H-^15^N HSQC spectra were collected on a 700 MHz Bruker Avance III spectrometer equipped with a 5 mm quadruple resonance QCI-P cryoprobe (^1^H, ^13^C, ^15^N, and ^31^P) with *z*-axis gradients. Order parameters (***S***^2^) were calculated from chemical exchange saturation transfer (CEST)-derived *R*_*1*_ and *R*_*2*_ relaxation rates and heteronuclear NOE values using FAST-Modelfree^44^ as previously described^42^.

### Secondary structure and thermal stability analysis of DJ-1

Reduced or Cys106-SO_3_^−^ DJ-1 was dialyzed into 10 mM potassium phosphate buffer pH = 7.0, diluted to a 12.5 μM monomer concentration, and placed in a 1 mm pathlength quartz cuvette for the acquisition of far UV circular dichroism (CD) spectra. CD spectra were acquired using a Jasco J-815 CD spectrometer (Jasco Inc., Easton, MD, USA) and normalized to total protein concentration as previously described^40^.

Thermal stabilities were measured using a scanning fluorimetry assay with Sypro Orange as previously reported^40^. First derivative plots of fluorescence as a function of temperature (d*F*/d*T*) were used to visualize the melting transition.

### Statistical analysis

We used one-way ANOVA to evaluate statistical differences among experimental groups. A value of *P* < 0.05 was considered significant. Data are shown as the mean ± s.e.m. from at least three independent experiments.

## Results

### DJ-1 overoxidation in ATII cells isolated from emphysema patients

**Fig. 1:**
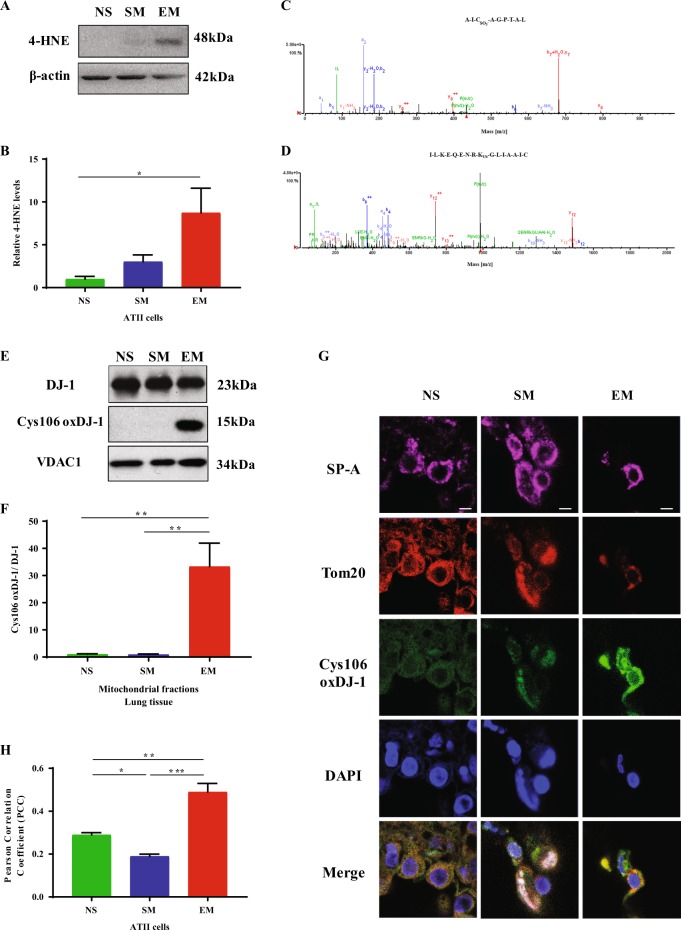
High oxidative stress induces Cys106-SO_3_^−^ within DJ-1 in ATII cells in emphysema patients. ATII cells were isolated from non-smokers, smokers, and emphysema patients. **a** 4-HNE expression was determined by western blotting. **b** Densitometric quantification is also shown. **c** Cys106-SO_3_^−^ within DJ-1 peptide AIC_SO3_-AGPTAL in freshly isolated ATII cells from emphysema patients as detected by mass spectrometry analysis. **d** Ubiquitination of lysine residue at position 99 within DJ-1 peptide ILKEQENRK_Ub_GLIAA in ATII cells obtained from individuals with emphysema. **e** DJ-1 expression and Cys106-oxidation within DJ-1 in mitochondrial fractions obtained from lung tissue of emphysema patients by western blotting. **f** Relative expression is also shown. **g** DJ-1 with oxidized Cys106 (green) in mitochondria identified by Tom20 expression (red) in ATII cells detected using SP-A antibody (magenta). Nuclei were stained with DAPI (blue). The scale bar is 5 μm. **h** Co-localization of DJ-1 with oxidized Cys106 and Tom20 (*N* = 3–7 lungs per group; *P < 0.05; **P < 0.01; ***P < 0.001). Data are shown as means ± s.e.m.

Oxidative stress was analyzed in freshly isolated ATII cells from non-smokers, smokers, and emphysema patients (Fig. 1a). We found higher 4-HNE expression, which is a marker of lipid peroxidation products, in ATII cells isolated from individuals with emphysema compared to control non-smokers (Fig. 1b). DJ-1 possesses a redox-sensitive Cys106, which is critical for its cytoprotective function and can be oxidized^13^. We immunoprecipitated DJ-1 to determine its oxidation status and Cys106 post translational modifications by mass spectrometry analysis in freshly isolated ATII cells. We detected Cys106-SO_3_^−^ within DJ-1 peptide AIC_SO3_AGPTAL in emphysema patients (Fig. 1c). Our data shows ubiquitination of lysine at position 99 within DJ-1 peptide ILKEQENRK_Ub_GLIAA (Fig. 1d). Of note, we did not detect these DJ-1 post translational modifications in ATII cells obtained from control non-smokers or smokers. Our results also indicate the presence of Cys106-SO_2_^−^ in IAAIC_SO2_AGPTALLAHEIG peptide in control smokers (Supplementary Fig. 1). We did not detect ubiquitination of this DJ-1 form by mass spectrometry analysis, which suggests that this active protein form is responsive to perturbations in redox homeostasis. Cys106-SO_2_^−^ within DJ-1 was not observed in ATII cells isolated from non-smokers. Identification of distinct Cys106 oxidation states in ATII cells from non-smokers, smokers, and emphysema patients establishes that the handling of DJ-1 does not result in artifactual overoxidation at Cys106, otherwise reduced Cys106 would not have been observed in control ATII cells. Our results indicate that high oxidative stress in ATII cells in emphysema may lead to DJ-1 overoxidation to the Cys106-SO_3_^−^ form.

### Oxidation of DJ-1 in mitochondria in ATII cells in emphysema

Since DJ-1 oxidation in ATII cells was detected, we wanted to determine the oxidation status of DJ-1 in mitochondrial fractions obtained from lung tissue of control non-smokers, smokers, and, emphysema patients. We found the presence of cleaved DJ-1 with oxidized Cys106 in this disease by western blotting (Fig. 1e, f). We were able only to see the trend in discrimination between the Cys-SO_2_^−^ and –SO_3_^−^ oxidation states using recombinant protein and this antibody (Supplementary Fig. 2). We performed immunohistofluorescence in human lung tissue using SP-A as a marker of ATII cells and Tom20 to identify mitochondria (Fig. 1g). We found higher levels of Cys106-oxidized DJ-1 in mitochondria in ATII cells in emphysema compared to controls, as shown by co-localization of oxidized DJ-1 with Tom20 (Fig. 1h). Our results suggest that oxidative stress in emphysema leads to DJ-1 oxidation in mitochondria, which may contribute to mitochondrial dysfunction.

### The cytoprotective role of DJ-1

**Fig. 2:**
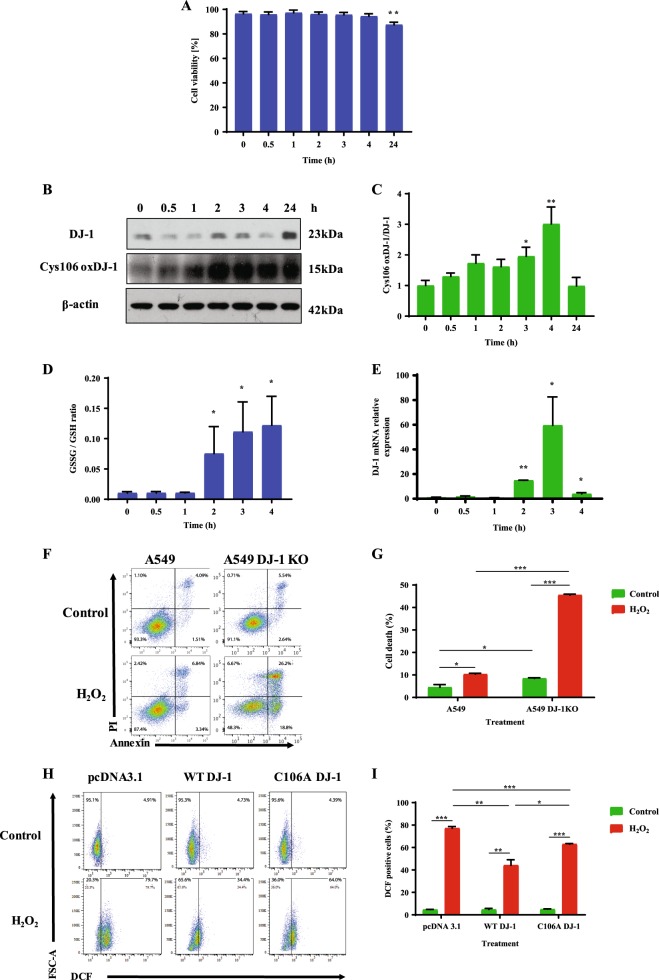
The cytoprotective role of DJ-1 against oxidative stress. A549 cells were treated with 1 mM H_2_O_2_ for indicated times. **a** Viability was estimated using Hoechst 33342 and PI double staining. **b** Expression of DJ-1 and Cys106-oxidized DJ-1 determined by western blotting. **c** Relative expression is also shown. **d** Oxidative stress analyzed by GSSG/GSH ratio. **e** DJ-1 mRNA expression was determined by RT-PCR. **f** Representative flow cytometry images using Annexin V and PI double staining in A549 cells and A549 cells with DJ-1 KO. **g** Quantification is also shown. **h** A549 cells with DJ-1 KO were transfected with pcDNA3.1, WT DJ-1, or C106A DJ-1 plasmids followed by treatment with 1 mM H_2_O_2_ for 2 h and analyzed using DCF staining. **i** Quantification is also shown. *N* = 3 replicates; *P < 0.05, **P < 0.01; ***P < 0.001. Data are shown as means ± s.e.m.

Wild-type A549 cell line was used as a model to determine the biological consequences of DJ-1 oxidation. We found that 1 mM H_2_O_2_ significantly decreased A549 cell viability after 24 h (Fig. 2a). To further gain an insight into the role of DJ-1, we determined its expression in whole A549 cell lysate by western blotting. We analyzed the expression of reduced DJ-1, and Cys106-oxidized DJ-1 (Fig. 2b). We found a statistically significant increase in the ratio of oxidized to reduced DJ-1 after 3 h, and 4 h treatment with 1 mM H_2_O_2_ (Fig. 2c).

We further focused on early events of cell injury induced by H_2_O_2_ to determine the mechanism of the DJ-1 function. Oxidative stress was determined in A549 cells treated with 1 mM of H_2_O_2_ at indicated times. GSSG/GSH ratio (Fig. 2d) indicates its increase in a time-dependent manner and we found statistically significant higher levels of oxidative stress after 2, 3, and 4 h. We also analyzed DJ-1 levels by RT-PCR in A549 cells. As shown in Fig. 2e, cell treatment with H_2_O_2_ increased DJ-1 mRNA expression after 2, 3, and 4 h. Our data suggests DJ-1-mediated cellular response under oxidative stress.

### The function of Cys106 within DJ-1

We generated A549 cells with DJ-1 knockout (A549 DJ-1 KO) using CRISPR-Cas9 strategy (Supplementary Fig. 3) to further determine the function of Cys106 within DJ-1. We treated A549 cells and A549 cells with DJ-1 KO with 1 mM H_2_O_2_ followed by flow cytometry analysis (Fig. 2f). We found significantly increased cell death in A549 cells with DJ-1 knockout compared to control cells (Fig. 2g). Moreover, cell death in DJ-1 KO A549 cells was increased after treatment with H_2_O_2_. Since these results indicate a cytoprotective role of DJ-1 against oxidative stress, we also wanted to determine the function of Cys106 in DJ-1-mediated cytoprotection. We transfected DJ-1 KO A549 cells with wild-type DJ-1 or C106A mutant DJ-1 plasmids. Transfection efficiency was confirmed using mCherry plasmid (Supplementary Fig. 4). DJ-1 overexpression in A549 cells was confirmed by western blotting (Supplementary Fig. 5). Since we detected high GSSG/GSH levels after cell treatment with 1 mM H_2_O_2_ (Fig. 2d), we also determined ROS generation using DCF. Transfection of A549 DJ-1 KO with WT DJ-1 followed by treatment with 1 mM H_2_O_2_ for 2 h significantly reduced ROS levels compared to control (Fig. 2h, i), but did not decrease cell death (Supplementary Fig. 6). On the other hand, we detected higher ROS generation in cells transfected with C106A mutant DJ-1 compared to WT DJ-1 plasmid. We also treated cells with 50 µM bleomycin to determine ROS generation using different trigger and we obtained a similar correlation (Supplementary Fig. 7). Our results indicate induction of DJ-1 expression in response to oxidative stress and the importance of Cys106 on DJ-1 cytoprotective function.

### Oxidative stress and DJ-1 ablation decreases mtDNA amount and increases mtDNA damage

**Fig. 3:**
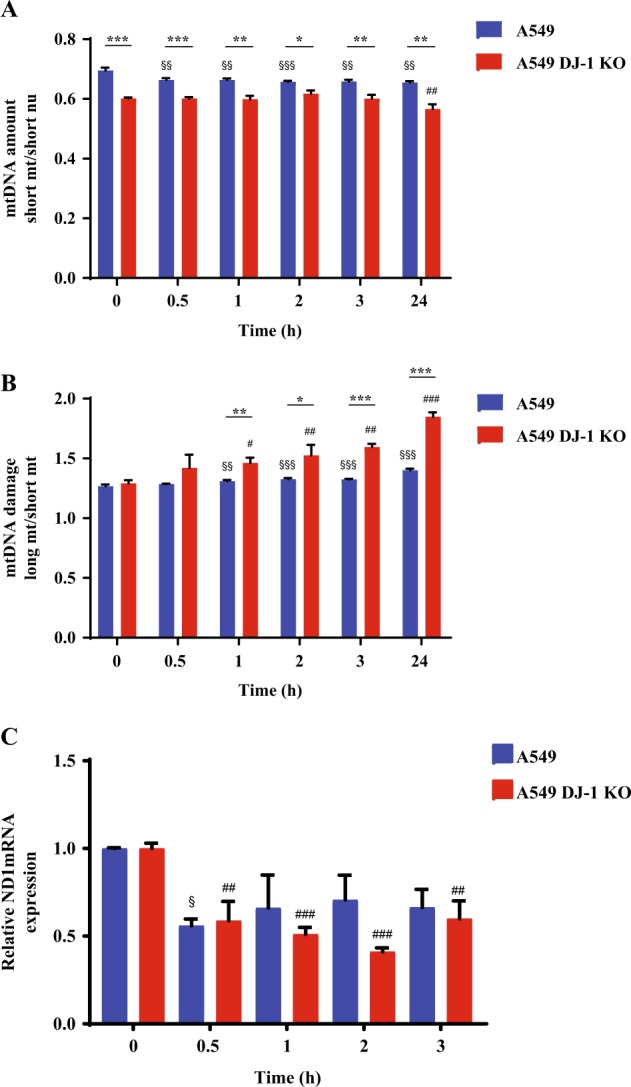
Mitochondrial dysfunction in A549 cells with DJ-1 knockout. A549 cells with DJ-1 KO were generated using CRISPR-Cas9 strategy. A549 cells and DJ-1 KO A549 cells were treated with 1 mM H_2_O_2_ for indicated times to determine mtDNA amount by qPCR (**a**), mtDNA damage using qPCR (**b**), and ND1 mRNA expression by RT-PCR (**c**). *P < 0.05, **P < 0.01, and ***P < 0.001. $*P* < 0.05, $$*P* < 0.01, and $$$*P* < 0.001 compared to control A549 cell line. #*P* < 0.05, ##*P* < 0.01, and ###*P* < 0.001 compared to control A549 cells with DJ-1 KO. *N* = 3 replicates. Data are shown as means ± s.e.m.

Control A549 cells and A549 DJ-1 KO cells were used for these experiments. We found that the treatment of A549 cells with H_2_O_2_ decreased mtDNA amount at early time points starting at 0.5 h (Fig. 3a). Surprisingly, we observed lower mtDNA amount in DJ-1 KO A549 cells at a later time point, i.e., after 24 h exposure. We also detected that DJ-1 KO A549 cells have slower growth compared to control cells (Supplementary Fig. 8).

Higher mtDNA damage was detected in A549 cells and DJ-1 KO A549 cells after 1, 2, 3, and 24 h treatment with H_2_O_2_ (Fig. 3b). We observed a significantly increased mtDNA damage in DJ-1 KO compared to control cells. In addition, we examined mitochondrial ND1 (NADH dehydrogenase 1) expression encoding for a subunit of the respiratory chain complex I. We found decreased ND1 levels in DJ-1 KO A549 cells treated with H_2_O_2_ for 0.5, 1, 2, and 3 h by RT-PCR (Fig. 3c). Control A549 cells treated with H_2_O_2_ had decreased ND1 expression only after 0.5 h treatment. This indicates lower mitochondrial recovery capacity in cells with DJ-1 deficiency in comparison with control cells. Our results suggest that DJ-1 KO decreases mtDNA amount and sensitizes cells to oxidative stress-induced mtDNA damage, which may contribute to delayed cell growth.

### DJ-1 localization under oxidative stress

**Fig. 4:**
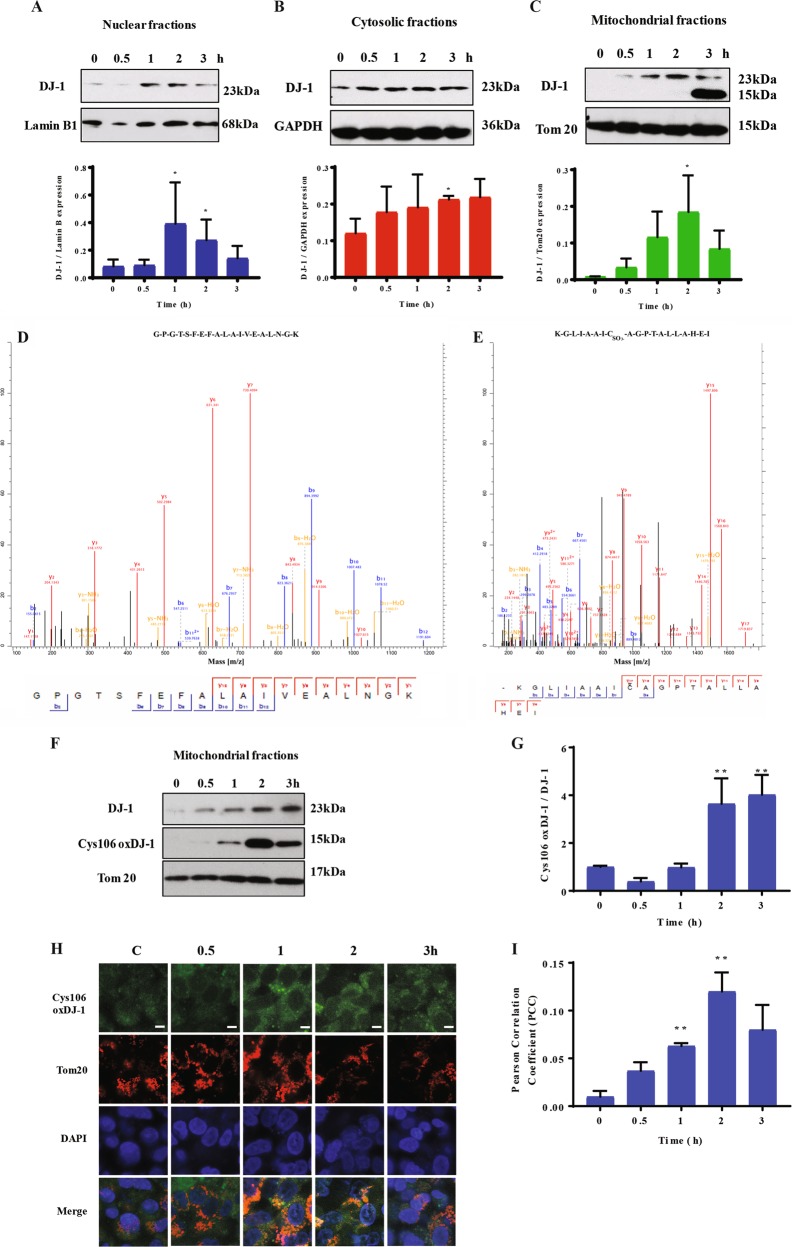
H_2_O_2_ induces Cys106-SO_3_^−^ DJ-1 formation in mitochondria. A549 cells were treated with 1 mM H_2_O_2_ for indicated times and DJ-1 expression was determined by western blotting in nuclear (**a**), cytosolic (**b**), and mitochondrial (**c**) fractions. Densitometric quantifications are also shown. Bands detected at 23 kDa and 15 kDa in mitochondrial fractions by western blotting were analyzed by mass spectrometry analysis. The presence of DJ-1 in the band at 23 kDa (**d**) and Cys106-SO_3_^−^ DJ-1 at 15 kDa (**e**) is shown. **f** Cys106-oxidized DJ-1 in mitochondrial fractions in A549 cells by western blotting. **g** Densitometric quantification is also shown. **h** Cys106-oxidized DJ-1 (green) in mitochondria identified by Tom20 (red) using immunocytofluorescence. Nuclei were stained with DAPI (blue). The scale bar is 5 μm. **i** Cys106-oxidized DJ-1 and Tom20 co-localization is shown. *N* = 3 replicates; *P < 0.05; **P < 0.01. Data are shown as means ± s.e.m.

We performed subcellular fractionation and determined the purity of nuclear, cytosolic, mitochondrial, and ER fractions by western blotting using lamin-B1, IKBα, COX IV, and PD1 antibodies, respectively, by western blotting (Supplementary Fig. 9). We observed a significant increase in DJ-1 levels in nuclear fractions after 1 h and 2 h treatment (Fig. 4a). DJ-1 expression was higher in cytosol only after 2 h treatment (Fig. 4b). We also found increased DJ-1 levels in mitochondrial fractions after 2 h (Fig. 4c). Our data indicate early DJ-1 upregulation in response to oxidative stress.

### Oxidation of DJ-1 at Cys106 to the sulfonate within mitochondria

We found a protein band at 15 kDa in addition to the DJ-1 band detected at 23 kDa in mitochondrial fraction after cell treatment with 1 mM H_2_O_2_ for 3 h (Fig. 4c). We performed mass spectrometry analysis to further identify this DJ-1 isoform. The stained 23 kDa or 15 kDa bands were excised, trypsinized, and subjected to LC-MS/MS analysis. Our results confirmed the presence of DJ-1 in both bands (Fig. 4d, e). We also detected Cys106-SO_3_^−^ DJ-1 only in the band at 15 kDa (Fig. 4e). This suggests that high oxidative stress induces both DJ-1 overoxidation and cleavage. Moreover, we isolated mitochondrial fractions from A549 cells treated with H_2_O_2_ to validate results obtained from mass spectrometry analysis and determine the levels of DJ-1 with oxidized Cys106 by western blotting (Fig. 4f). The highest expression of Cys106-oxidized DJ-1 was observed at 2 h and 3 h (Fig. 4g). Furthermore, we performed immunohistofluorescence to analyze the expression of Cys106-oxidized DJ-1 in mitochondria after cell treatment with H_2_O_2_ (Fig. 4h). We detected higher co-localization of Cys106-oxidized DJ-1 and Tom20 after 1 h and 2 h exposure (Fig. 4i)_._ Our results suggest that oxidative stress induces DJ-1 overoxidation and cleavage in mitochondria.

### Formation of Cys106-SO_3_^−^ destabilizes DJ-1 in vitro

**Fig. 5:**
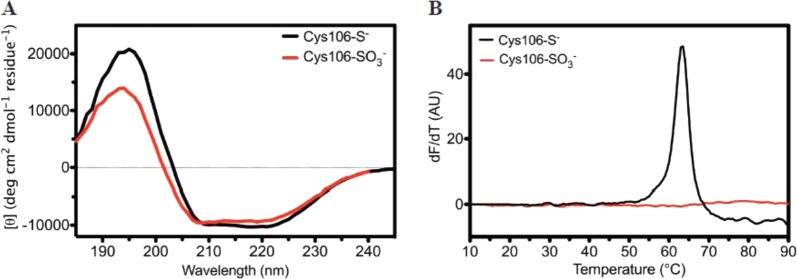
Cys106-SO_3_^−^ DJ-1 formation preserves secondary structure but destabilizes the protein. **a** Circular dichroism (CD) spectra are shown for reduced (black) and Cys106-SO_3_^−^ DJ-1 (red) DJ-1. Formation of Cys106-SO_3_^−^ results in a minor decrease in secondary structural content. The *y*-axis shows per-residue molar ellipticity ([θ]). **b** The Thermofluor scanning fluorimetry assay was used to measure the melting temperature (Tm) of reduced (black) and Cys106-SO_3_^−^ (red) DJ-1. The first derivative of the fluorescence as a function of temperature (d*F*/d*T*) is shown. Reduced DJ-1 has a Tm of 63 °C, while there is no measured transition for Cys106-SO_3_^−^ DJ-1, indicating substantial destabilization

Recombinant DJ-1 was prepared in the reduced and predominantly Cys106-SO_3_^−^ forms as verified by electrospray mass spectrometry (Supplementary Fig. 2). Circular dichroism (CD) spectroscopy indicates that Cys106-SO_3_^−^ DJ-1 has modestly diminished secondary structure compared to the reduced form (Fig. 5a), consistent with prior reports^25,45^. Thermal stability analysis using the Thermofluor assay^46^ shows that formation of Cys106-SO_3_^−^ dramatically reduces the magnitude of the thermal unfolding transition, indicating global destabilization of the protein (Fig. 5b). This destabilization contrasts sharply with the 12 °C stabilization of DJ-1 observed upon Cys106-SO_2_^−^ formation^47^, indicating that these two similar oxidative modifications at Cys106 have disparate effects on DJ-1 stability.

**Fig. 6:**
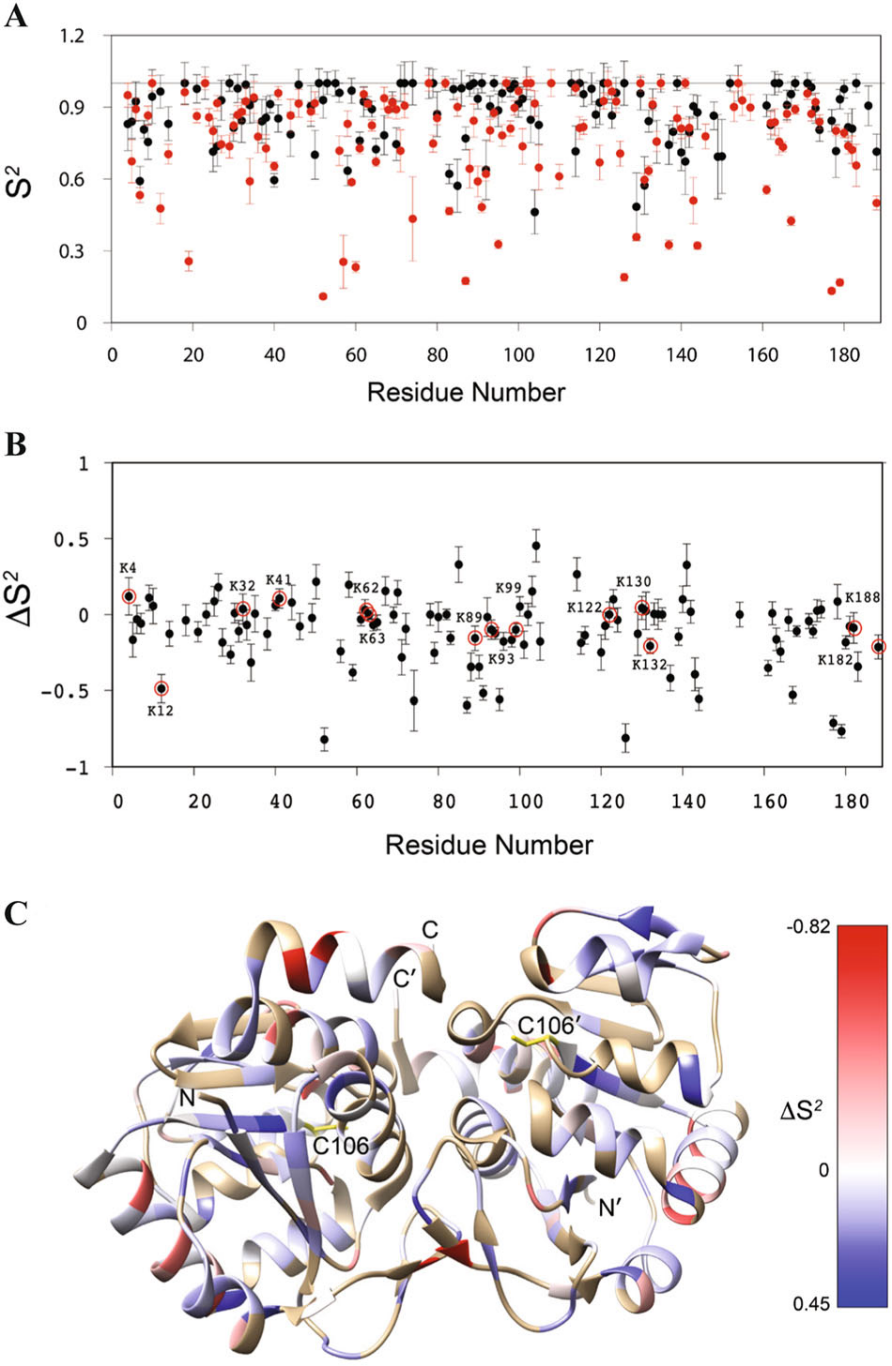
NMR relaxation analysis of picosecond–nanosecond (ps–ns) timescale dynamics in Cys106-SO_3_^−^ DJ-1. **a** The backbone 1H-15N amide bond vector order parameters (S2) in reduced (black) and Cys106-SO_3_^−^ DJ-1 (red). S2 range from 1 for immobile residues on the ps–ns timescale to 0 for highly mobile bond vectors with no preferred orientation. Cys106-SO_3_^−^ formation results in a marked lowering of S2 values in several regions of DJ-1. **b** The ΔS2 values calculated as: ΔS2 = S2(Cys106-SO_3_^−^)- S2(reduced) for each residue. Negative values indicate residues that become more mobile in Cys106-SO_3_^−^ DJ-1 and positive values indicate residues that become less mobile. Lysine residues are circled and labeled. **c** The ΔS2 values onto the ribbon diagram of the DJ-1 dimer. Red values indicate increased mobility and blue values indicated decreased mobility in Cys106-SO_3_^−^ DJ-1. The core of the dimer experiences a slight decrease in mobility upon formation of Cys106-SO_3_^−^, while the solvent-exposed regions have strongly increased mobility

We used NMR to measure the picosecond–nanosecond (ps–ns) timescale amide backbone dynamics of DJ-1. Broadly consistent with the CD and thermal stability analysis, we found that Cys106-SO_3_^−^ caused several areas of the protein to become more mobile (lower S^2^ order parameters), although other areas were less dynamic (higher S^2^ order parameters) (Fig. 6a). We evaluated these backbone amide order parameters to determine how increased flexibility of DJ-1 with Cys106-SO_3_^−^ affects lysine residues. Lysine residues of the substrate protein play a role in ubiquitination, which targets misfolded proteins for degradation^48,49^. DJ-1 has 16 lysine residues and we found that this protein overoxidation increased backbone ps–ns timescale mobility of several lysine residues compared to reduced DJ-1 form, including K12, K89, K93, K99, K132, K182, and K188 (Fig. 6b). These results are in agreement with our findings showing DJ-1 ubiquitination at K99 in ATII cells in emphysema (Fig. 1d). By mapping the difference order parameters (ΔS^2^) onto the structure of DJ-1, it was apparent that Cys106-SO_3_^−^ formation increased ps–ns mobility of external portions of the protein, although areas in the core of the protein were slightly less mobile in Cys106-SO_3_^−^ DJ-1 than in the reduced protein (Fig. 6c). Altogether, our data indicate that Cys106-SO_3_^−^ formation destabilizes DJ-1 and renders solvent-exposed secondary structural elements more flexible the ps–ns timescale, which may affect this protein cytoprotective function.

## Discussion

DJ-1 is a multifunctional protein that protects cells against oxidative stress^50^. Its oxidation occurs predominantly through direct oxygen addition to the reactive Cys106 residue, which can form multiple oxidized species. In this study, we discovered that Cys106 is preferentially overoxidized to the sulfonate (Cys106-SO_3_^−^) in ATII cells in emphysema, which can affect DJ-1 cytoprotective function.

Oxidative stress has been shown to contribute to emphysema pathogenesis, which is characterized by alveolar wall destruction^3,51^. There is also an oxidant–antioxidant imbalance in this disease^52^. We have previously reported lower DJ-1 expression in ATII cells isolated from heavy smokers^26^. Of note, we found that DJ-1 overexpression restored its cytoprotective function in these individuals^26^. Here, we found higher oxidative stress in ATII cells isolated from emphysema patients in comparison with control smokers and non-smokers. Cys106 is the most sensitive among three cysteine residues in human DJ-1 to oxidative stress and regulates DJ-1 function as a ROS sensor^17^. However, very high oxidative stress causes overoxidation of Cys106 to the sulfonate^13^. Our data suggests that the highly oxidized Cys106-SO_3_^−^ isoform of DJ-1 is present in ATII cells in this disease but was not detected in cells isolated from control non-smokers or smokers, where we observed only reduced or Cys106-SO_2_^−^ DJ-1, respectively. However, chemical tools with omics technologies and advanced computational methods may be required to further study oxidized DJ-1 forms. Our results suggest that the overoxidation of DJ-1 decreases its cytoprotective function in emphysema. It has been shown that Cys106-SO_3_^−^ represents an irreversible overoxidized DJ-1 form^53^. Cys106-SO_3_^−^ DJ-1 was also identified in the blood and erythrocytes of patients with Parkinson disease, suggesting a broader connection between highly oxidized DJ-1 and disease pathogenesis^54,55^.

This irreversibly overoxidized DJ-1 form is a subject to degradation^24^. The molecular basis of Cys106-SO_3_^−^-induced destabilization of DJ-1 and the cellular mechanisms, leading to the degradation of highly oxidized DJ-1 are still not understood. Using NMR relaxation measurements, we found that the formation of Cys106-SO_3_^−^ leads to an increase in ps–ns timescale mobility of several regions on the surface of DJ-1. In addition, the formation of Cys106-SO_3_^−^ caused the loss of any observed thermal melting transition for DJ-1, despite having only a minor loss of secondary structure. The presence of well-resolved resonances in the ^1^H-^15^N HSQC NMR spectrum of Cys106-SO_3_^−^ DJ-1 establishes conclusively that this protein form is well-folded at 37 °C, however, it appears to be markedly destabilized based on its melting behavior. The NMR relaxation experiments performed here are only sensitive to ps–ns timescale dynamics, and it is possible that longer timescale dynamics are more severely perturbed in Cys106-SO_3_^−^ DJ-1. We propose that the increased dynamics and decreased stability of Cys106-SO_3_^−^ DJ-1 is the molecular cause of the increased ubiquitination of DJ-1 that we observed under high oxidative burden in ATII cells in emphysema, as well as its cleavage in the mitochondria. In addition, increased flexibility of Cys106-SO_3_^−^ DJ-1 explains why this highly oxidized isoform does not crystallize, while both reduced and Cys106-SO_2_^−^ DJ-1 forms crystals readily. It has been reported that DJ-1 with Cys106-SO_3_^−^ can facilitate DJ-1 interactions with ubiquitin ligases^56^, which can contribute to loss of DJ-1 cytoprotective function. Moreover, other observations suggest that oxidized forms of DJ-1 and other redox-sensitive proteins are readily degraded^56^, consistent with our findings. Further studies are required to determine how the redox signaling of subcellular compartments and extracellular microenvironment influence DJ-1 post translational modifications.

Our results correlate with a combined computational and in vitro evaluation of the effect of Cys106 oxidation on DJ-1 structure and dynamics, and both approaches note an increase in DJ-1 mobility in several regions as a consequence of Cys106-SO_3_^−^ formation^25^. However, a notable difference is that Kiss et al.^25^ found no change in DJ-1 thermal stability in going from Cys106-SO_2_^−^ to -SO_3_^−^ using differential scanning calorimetry, where we observe a complete loss of a melting transition for Cys106-SO_3_^−^ DJ-1 using the Thermofluor assay. These two experimental methods have typically delivered comparable melting temperature (*T*_m_) values for DJ-1^47^, so the cause for this discrepancy is unclear. Our results indicate that the redox-sensitive Cys106 residue is subject to distinct, physiologically relevant modifications that biphasically regulate the cytoprotective function of DJ-1 through changes in protein flexibility, stability, and interaction potential in the critical Cys106 pocket.

We used A549 cells as a model to further determine the cellular consequences of Cys106-SO_3_^−^ formation in DJ-1 under controlled conditions using 1 mM H_2_O_2_ as previously reported^31–34^. We found high oxidative stress in A549 cells treated with H_2_O_2_ as determined by GSSG/GSH ratio. GSH is the most abundant small molecule thiol in cells and is a key factor in the defense system against ROS generation^57,58^. GSSG is the dominant oxidized state of GSH^59^. We further determined the function of DJ-1 using A549 cells with DJ-1 KO generated using the CRISPR-Cas9 strategy. Our results indicate that DJ-1 ablation decreases cell viability after treatment with H_2_O_2_ compared to the control A549 cell line. Transfection of A549 cells with wild-type DJ-1 reduced ROS levels and restored cytoprotection. Interestingly, cell transfection with C106A DJ-1 also reduced ROS level; however to a lesser degree. This indicates that C106A mutation does not completely abolish cytoprotective function of DJ-1, which is in agreement with other studies^60,61^. Altogether, our results suggest the critical role of DJ-1 in sensing and activating cellular responses to ROS. These observations are in agreement with our previously published study showing that DJ-1 KO mice exposed to cigarette smoke have higher pro-inflammatory response compared to wild-type mice^27^. Other studies have shown that embryonic stem cells deficient in DJ-1 display increased sensitivity to oxidative stress^62^. Moreover, the accumulation of ROS in toxin-treated cells contributed to damage that ultimately led to apoptotic death.

DJ-1 is localized in the cytoplasm, nuclei, and mitochondria^13^ and is translocated to cell compartments without localization sequence signal^50,63^. This suggests that post translational modifications of this protein such as oxidation can affect its subcellular localization. However, mechanisms underlying these processes and DJ-1’s cytoprotective function, remain to be fully elucidated. Indeed, first, we found DJ-1 with Cys106-SO_3_^−^ in mitochondria after cell treatment with H_2_O_2_ and second, we detected cleavage of this DJ-1 form by mass spectrometry analysis. The Cys106-oxidation and cleavage of DJ-1 occurred in A549 as an early event of oxidative stress. However, expression of Cys106-overoxidized and cleaved DJ-1 persisted in emphysema patients. This accumulation may be explained by proteostasis-imbalance in chronic obstructive pulmonary disease as reported by Min et al. ^64^. Additionally, we found greater mtDNA damage and decreased ND1 expression in DJ-1 KO A549 cells treated with H_2_O_2_ compared to control cells. Of note, DJ-1 deficiency reduced mtDNA amount and cell growth. It was reported that mitochondrial dysfunction and a low mitochondrial amount can contribute to slow cell growth^65^. The delay in eliminating damaged mtDNA may also be explained by a defect in mitochondrial quality control (e.g., mitophagy)^66^. It has been reported that in the pathobiology of lung diseases, impaired mitochondria contribute to reduced mitochondrial mass and ATP production^67^. ROS can also induce mtDNA damage and mitochondrial dysfunction, leading to cell death^68^. Our findings showing the formation of Cys106-SO_2_^−^ within DJ-1 in ATII cells in smokers also suggest that this active DJ-1 form may have a cytoprotective role through the elimination of ROS-mediated damaged mitochondria. In conclusion, we show conformational changes of Cys106-SO_3_^−^ DJ-1, which suggests a loss of DJ-1 cytoprotective activity in ATII cells in emphysema. Further studies are required to determine the function of DJ-1 in mitochondria in lung diseases.

## Supplementary information

Supplementary materials and methods

Supplementary results
