# Supplementary material for: The effect of cysteine oxidation on DJ-1 cytoprotective function in human alveolar type II cells
Source: Cell Death Dis. 2019 Sep 2;10(9):638. doi: 10.1038/s41419-019-1833-5 (PMC6717737; doi:10.1038/s41419-019-1833-5)
Supplement: Supplementary file 1 — Supplementary materials and methods [file 41419_2019_1833_MOESM1_ESM.pdf]

Supplementary Materials and Methods

The effect of cysteine oxidation on DJ-1 cytoprotective function in human alveolar type II cells

Karim Bahmed[1,2#], Samia Boukhenouna[1,2#], Loukmane Karim[1,2], Tessa Andrews[3], Jiusheng Lin[3], Robert Powers[3,4,5], Mark A. Wilson[3,5], Chih-Ru Lin[1,2], Elise Messier[6], Nichole Reisdorph[6], Roger L. Powell[6], Hsin-Yao Tang[7], Robert J. Mason[6], Gerard J. Criner[1,2], Beata Kosmider[1,2,8,*]

[1]Department of Thoracic Medicine and Surgery, Temple University, Philadelphia, PA 19140

[2]Center for Inflammation, Translational and Clinical Lung Research, Temple University, Philadelphia, PA 19140

[3]University of Nebraska, Lincoln, NE 68588

[4]Nebraska Center for Integrated Biomolecular Communication, University of Nebraska, Lincoln, NE 68588

[5]Redox Biology Center, University of Nebraska, Lincoln, NE 68588

[6]National Jewish Health, Denver, CO, 80206

[7]The Wistar Institute, Philadelphia, PA 19104

[8]Department of Physiology, Temple University, Philadelphia, PA 19140

# These authors contributed equally to this work

* Correspondence:
Beata Kosmider, PhD or Karim Bahmed, PhD
Department of Thoracic Medicine and Surgery
Center for Inflammation, Translational and Clinical Lung Research
Temple University
3500 N. Broad Street
Philadelphia, PA 19140
E-mail: beata.kosmider@temple.edu

Supplementary Table 1. Primers used to analyze mRNA expression by RT-PCR, mtDNA amount and mtDNA damage by qPCR.

Gene		Primer's sequence (5'-3')
GAPDH	F	GGAGCGAGATCCCTCCAAAAT
	R	GGCTGTTGTCATACTTCTCATGG
DJ-1	F	GTAGCCGTGATGTGGTCATT
	R	CTGTGCGCCCAGATTACC
ND1	F	CCGAATCACCTGACGGTTCA
	R	TCTGGCTGCTTCTGAATCCC
Short mtDNA fragment	F	GAATTAACTAGAAATAACTTTGCAAGG
	R	CCAGCTATCACCAGGCTC
Long mtDNA fragment	F	ACAAAGAACCCTAACACCAGC
	R	TGTTGAGCTTGAACGCTTTC
Short nuclear DNA fragment	F	TGCTGTCTCCATGTTTGATGTATCT
	R	TCTCTGCTCCCCACCTCTAAGT
