# Supplementary material for: The effect of cysteine oxidation on DJ-1 cytoprotective function in human alveolar type II cells
Source: Cell Death Dis. 2019 Sep 2;10(9):638. doi: 10.1038/s41419-019-1833-5 (PMC6717737; doi:10.1038/s41419-019-1833-5)
Supplement: Supplementary file 2 — Supplementary results [file 41419_2019_1833_MOESM2_ESM.pdf]

Supplementary Results

The effect of cysteine oxidation on DJ-1 cytoprotective function in human alveolar type II cells

Karim Bahmed[1,2#], Samia Boukhenouna[1,2#], Loukmane Karim[1,2], Tessa Andrews[3], Jiusheng Lin[3], Robert Powers[3,4,5], Mark A. Wilson[3,5], Chih-Ru Lin[1,2], Elise Messier[6], Nichole Reisdorph[6], Roger L. Powell[6], Hsin-Yao Tang[7], Robert J. Mason[6], Gerard J. Criner[1,2], Beata Kosmider[1,2,8,*]

[1]Department of Thoracic Medicine and Surgery, Temple University, Philadelphia, PA 19140

[2]Center for Inflammation, Translational and Clinical Lung Research, Temple University, Philadelphia, PA 19140

[3]University of Nebraska, Lincoln, NE 68588

[4]Nebraska Center for Integrated Biomolecular Communication, University of Nebraska, Lincoln, NE 68588

[5]Redox Biology Center, University of Nebraska, Lincoln, NE 68588

[6]National Jewish Health, Denver, CO, 80206

[7]The Wistar Institute, Philadelphia, PA 19104

[8]Department of Physiology, Temple University, Philadelphia, PA 19140

# These authors contributed equally to this work

* Correspondence:
Beata Kosmider, PhD
Department of Thoracic Medicine and Surgery
Center for Inflammation, Translational and Clinical Lung Research
Temple University
3500 N. Broad Street
Philadelphia, PA 19140
E-mail: beata.kosmider@temple.edu

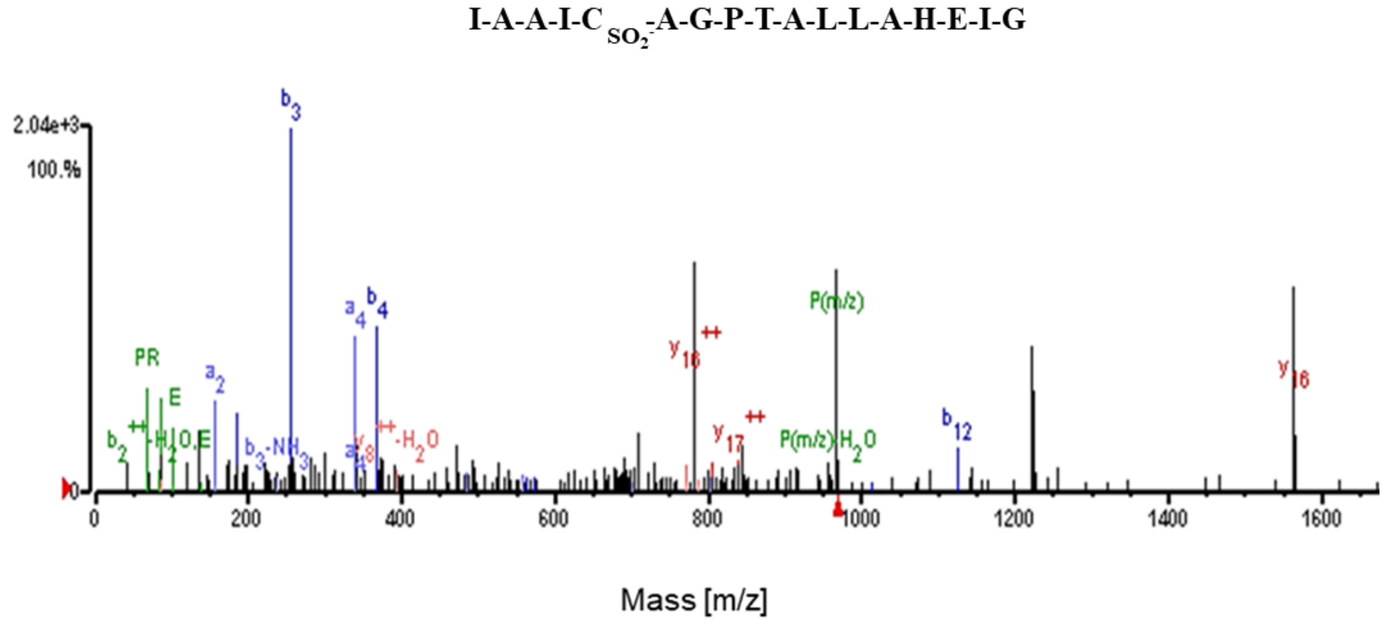

Supplementary Fig. 1. Cys106-SO_2^- within DJ-1 in freshly isolated ATII cells from smokers as detected by mass spectrometry analysis. Cys106-SO_2^- was detected in DJ-1 peptide IAAICAGPTALLAHEIG (N=3 lungs).

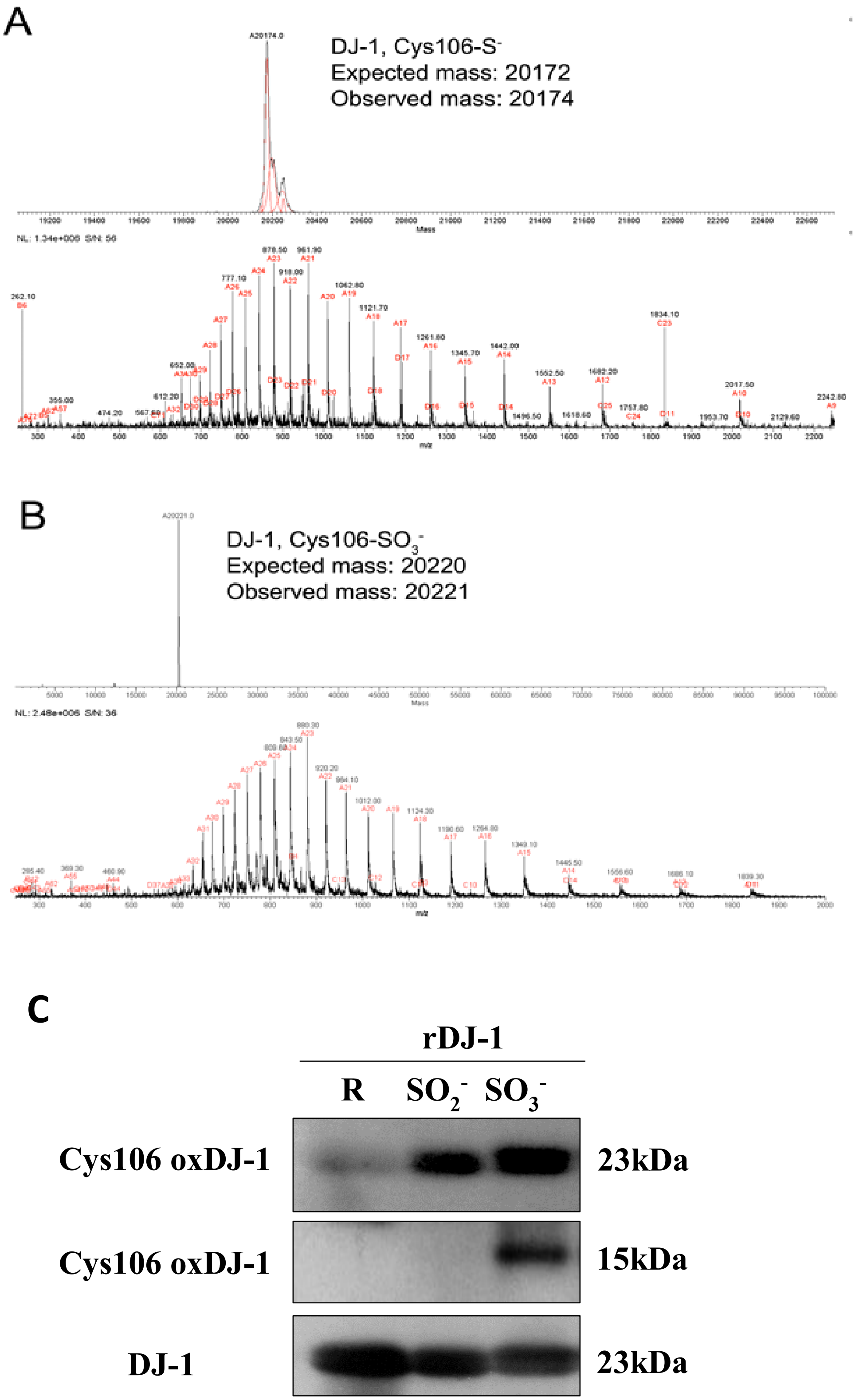

Supplementary Fig. 2. Intact mass spectrometry of recombinant DJ-1 proteins. LC-MS electrospray mass spectrometry was performed with a Dionex U3000 nano LC system and an ABSCIEX Q-TRAP 4000 mass spectrometer. **A** - Cys106-reduced recombinant DJ-1. **B** - Cys106-SO_3^- DJ-1 was prepared as described in the Materials and Methods section. Data were collected using LC-MS electrospray mass spectrometry with a Dionex U3000 nano LC system and an ABSCIEX Q-TRAP 4000 mass spectrometer. **C** - DJ-1 oxidation status was analyzed by Western blotting using antibody against oxidized DJ-1 (R – reduced, N=3).

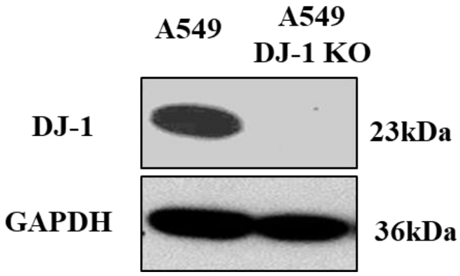

Supplementary Fig. 3. A549 cells with DJ-1 knockout generated using CRISPR-Cas9 strategy. A549 cell line with DJ-1 knockout was generated as described in Materials and Methods section. The absence of DJ-1 expression was confirmed by Western blotting.

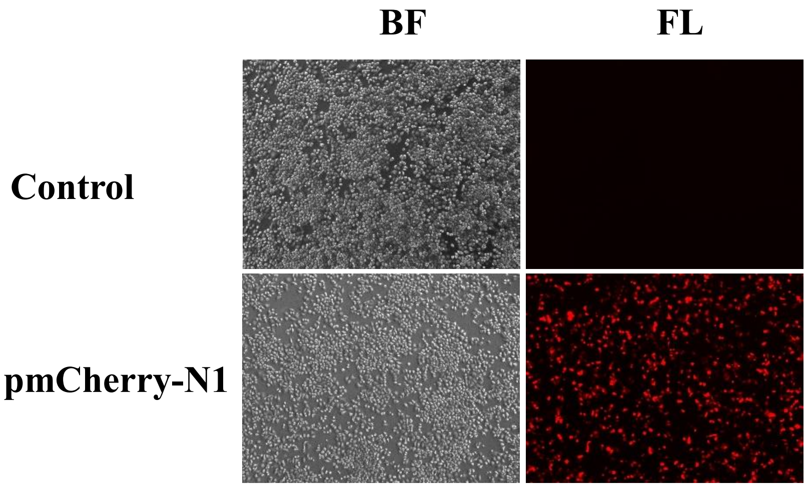

Supplementary Fig. 4. A549 cell transfection with pmCherry-N1 plasmid. Representative micrographs of the bright field (BF) and fluorescence (FL; 561 nm, red) channels of control A549 cells and cells transfected with pmCherry-N1 plasmid for 24h (N=3, magnification 20x).

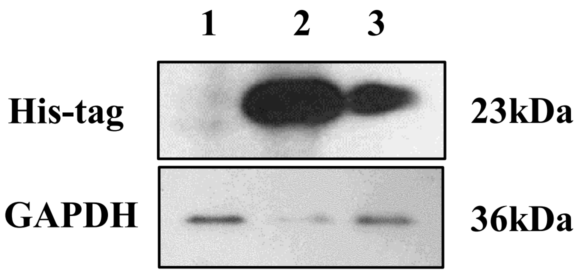

Supplementary Fig. 5. A549 cells transfected with DJ-1 plasmids. Cell transfection with plasmids was confirmed using His-tag antibody by Western blotting. Lane 1 – control A549 cells; lane 2 – cells transfected with wild type DJ-1 plasmid; lane 3 – cells transfected with C106A DJ-1 mutant construct.

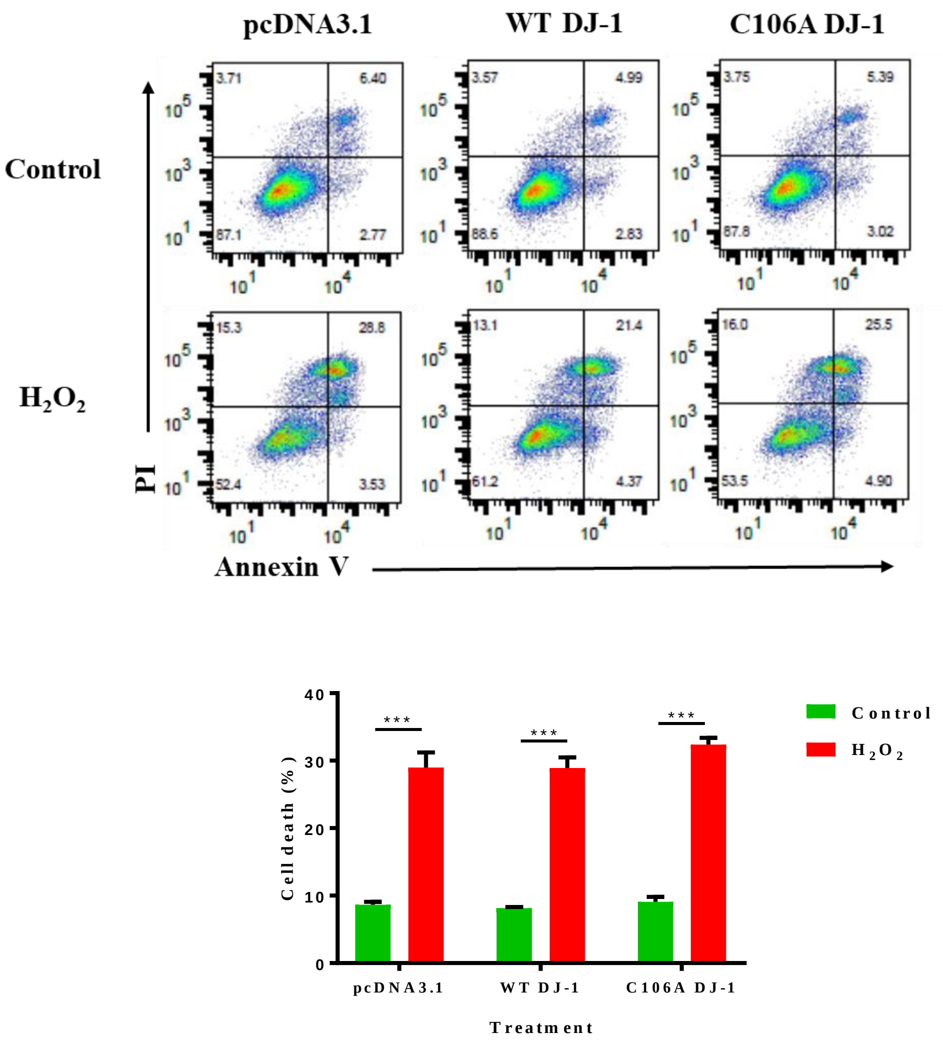

Supplementary Fig. 6. **DJ-1 knockout A549 cell treatment with H_2O_2.** A549 cells were treated with 1mM H_2O_2 for 24h. Viability was estimated using Annexin V and PI double staining. Representative flow cytometry images and relative expression is also shown. N = 3, *** $P < 0.001$. Data are shown as means ± s.e.m.

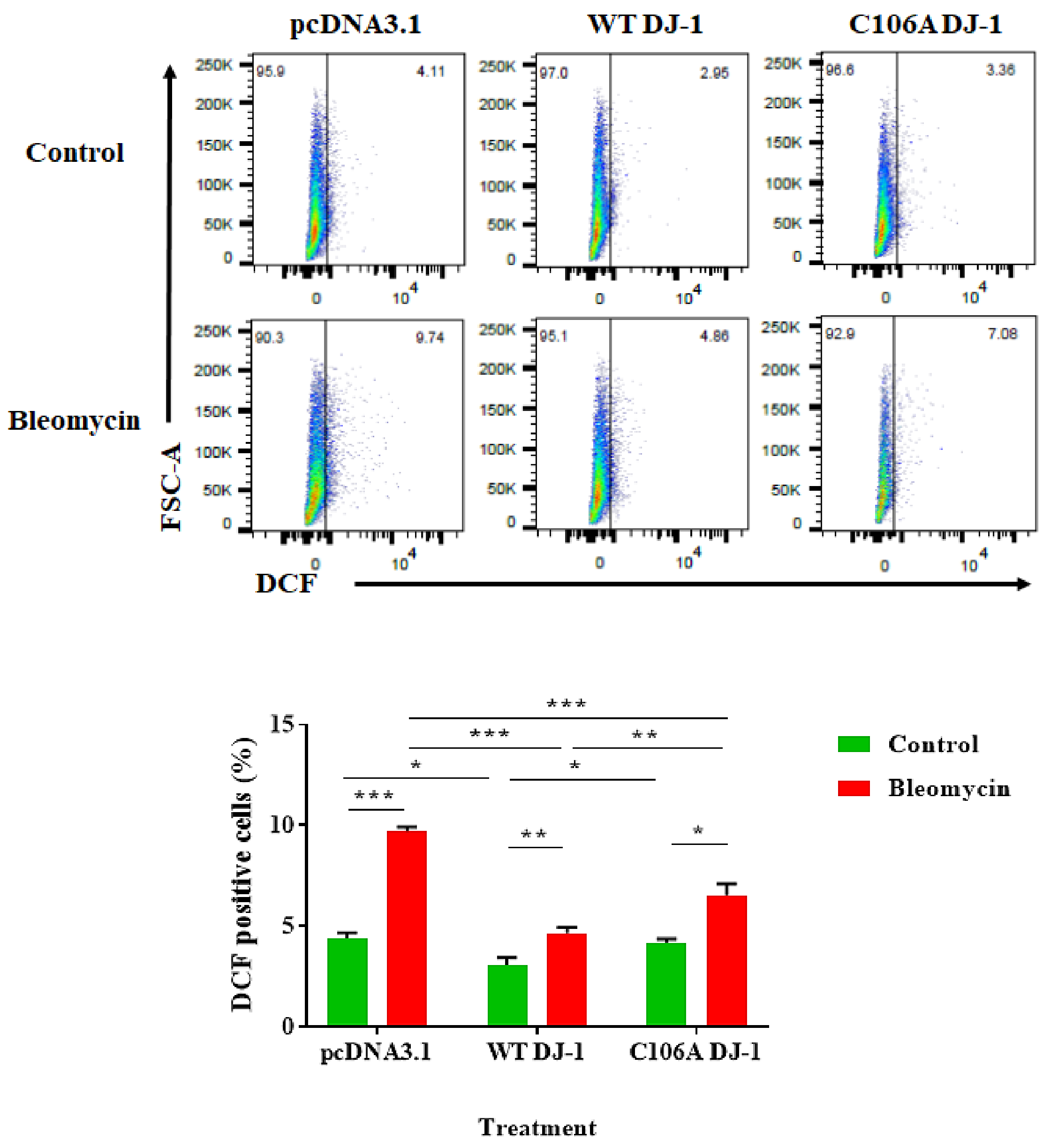

Supplementary Fig. 7. ROS generation in A549 cells with DJ-1 knockout. Cells were transfected with WT DJ-1 plasmid or C106A DJ-1 mutant construct as described in the Materials and Methods section followed by treatment with 50 µM bleomycin for 2h and analysis using DCF staining by flow cytometry. Quantification is also shown. N = 3 replicates; *P < 0.05, ** P < 0.01; ***P < 0.001. Data are shown as means ± s.e.m.

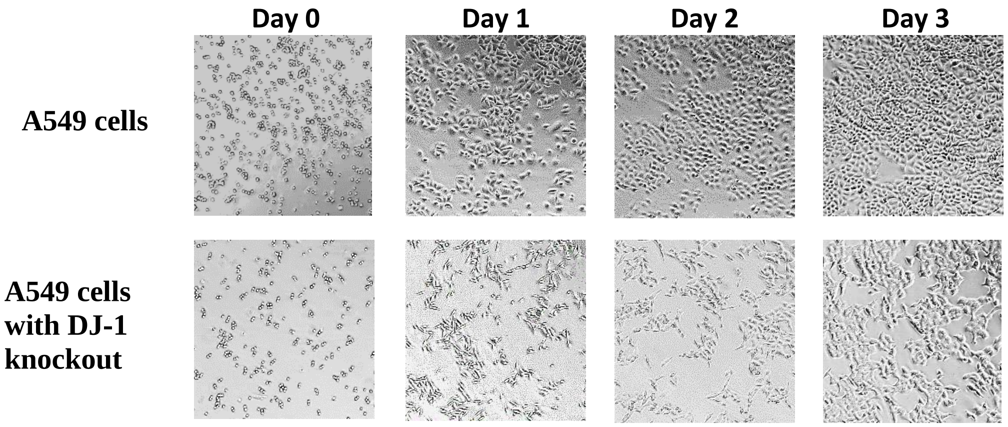

Supplementary Fig. 8. Decreased A549 cell growth with DJ-1 knockout. $4x10^4$ cells per 1.9 cm^2 were plated and cultured for 3 days. Representative pictures are shown (N=3 replicates).

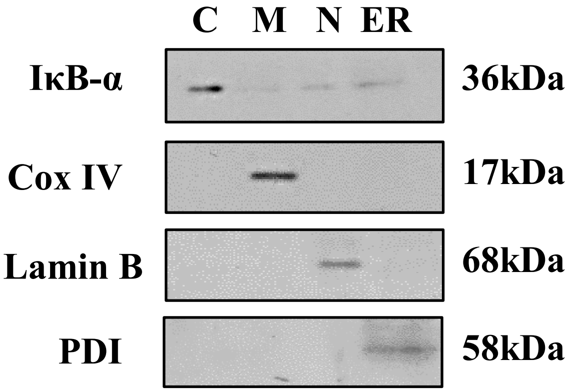

Supplementary Fig. 9. Purity of subcellular fractions obtained from A549 cells. Subcellular fractions were isolated from A549 cells as described in the Materials and Methods section. The purity of cytosolic (C), mitochondrial (M), nuclear (N) and endoplasmic reticulum (ER) fractions was confirmed by Western blotting (N=3 replicates).
